# A positive psychology framework for why people use substances: Implications for treatment

**DOI:** 10.3389/fpsyg.2022.1017186

**Published:** 2022-09-28

**Authors:** Bryant M. Stone

**Affiliations:** Department of Psychological and Behavioral Sciences, Institute of Psychiatry, Medical University of South Carolina, Charleston, SC, United States

**Keywords:** substance use, substance use disorders, PERMA, positive psychology, treatments

## Introduction

There is no doubt that the prevalence of substance use disorders is a public health crisis, with estimates in the US suggesting that over 20 million people meet the criteria for a substance use disorder (Substance Abuse Mental Health Services Administration, 2019) and involve a wide range of individuals, including veterans, physicians, athletes, adolescents, and individuals with mental and physical health concerns (Cuffel, 1996; Aarons et al., 2001; Oreskovich et al., 2015; Gil et al., 2016; Teeters et al., 2017; Yusufov et al., 2019). Researchers have found evidence of many neurological, affective, and behavioral theories on substance use with robust evidence (Hesselbrock et al., 1999; de Wit and Phan, 2010; Berridge and Robinson, 2016). Still, researchers have yet to integrate major theories of positive psychology into the substance use literature. The PERMA model (i.e., a model that suggests that positive emotions, engagement, relationships, meaning, and accomplishments explain variations in happiness; Seligman, 2011) may provide a valuable perspective for explaining why individuals use substances. I argue that when individuals are unable to achieve the PERMA domain that helps individuals experience an authentically happy life without substances, some individuals find success in meeting these domains with substances, which explains why it is so difficult for those with substance use disorder to abstain or reduce their use (see Table 1 for examples). In the current article, I further hypothesize and present research on why individuals use substances through the PERMA model and discuss how we can use this model in treatment.

**Table 1:** Examples of non-substance-related and substance-related behaviors from a PERMA model.

**Domain**	**Positive emotions**	**Engagement**	**Relationships**	**Meaning/Purpose**	**Accomplishments**
Non-substance-related	Exercising or eating good food	Going on a nature walk	Bonding with people in the community	Writing fictional books or stories	Giving a public speech
Substance-related	Smoking methamphetamines to feel happy	Watching a movie on MDMA	Bonding with people who use the same substances	Learning how to properly store and use a substance	Using alcohol before giving a public speech to feel less anxious

Examples are not exhaustive.

### Positive emotions

The positive emotions component refers to the experience of activities and situations resulting in pleasure or behavioral rewards in substance use (Seligman, 2011). Researchers have long implicated behavioral reward processes involved in substance use behaviors (i.e., behaviors that offer immediate pleasure or satisfaction from using substances; Berridge and Robinson, 2016). Further, more recent research has applied behavioral economics to substance use disorders, which suggests that substance use behaviors are reinforced by the availability and perceived immediate benefit of substance-related rewards despite the long-term consequences of such rewards (Murphy et al., 2012; Bickel et al., 2014; Teeters and Murphy, 2015; Murphy and Dennhardt, 2016; Field et al., 2020). Thus, substances provide immediate behavioral rewards, or pleasurable experience, that often results in positive emotions.

The PERMA model suggests that the experience of positive emotions is essential to living an authentically happy life. However, when individuals have difficulty experiencing positive emotions from non-substance-related activities (e.g., exercising), they may seek to experience positive emotions from substances. In treatments, reorienting clients and promoting behavioral activation to activities that may elicit positive emotions without the use of substances may be able to compensate for the experience of positive emotions that clients have begun to depend on from substances. Changing the source of positive emotions is challenging but not impossible. It is an essential treatment component of contingency management (see Prendergast et al., 2006 for a meta-analysis), which has shown promising effects. As such, clinicians must reorient clients to the experience of positive emotions mainly sourced from non-substance-related activities; otherwise, individuals may begin to rely on substances for their experiences of positive emotions.

### Engagement

Engagement in the PERMA model refers to experiences of being present and actively enthralled by pleasurable activities (Seligman, 2011). When individuals cannot experience engagement because of their living situations (e.g., limited free time from working excessive hours to make ends meet), mental health concerns (e.g., anhedonia in depression), or disabilities (e.g., disabilities making it challenging or dangerous to leave the home), which are all known risk factors for substance use (e.g., Cuffel, 1996; Moore and Li, 1998; Silverman et al., 2019), individuals may seek out substances to enhance experiences and promote engagement. Researchers have documented that many substances can enhance experiences, including nicotine, cannabis, LSD (i.e., acid), amphetamines, and MDMA (i.e., ecstasy or molly; Gilbert and Gilbert, 1998; Zeiger et al., 2010; Holze et al., 2020). Many individuals report a significant pleasurable enhancement in food consumption, sex, music, and mystical experiences in nature-related activities. As such, substances are a quick way for individuals to enhance engagement in activities, even mundane activities, when natural engagement in pleasurable activities is not sufficient or accessible.

Similar to experiencing positive emotions from non-substance-related rewards, clinicians may consider teaching clients skills to enhance engagement without using substances through two means: First, problem-solving with clients to increase the amount of time and accessibility of pleasurable experiences, which is akin to a behavioral activation approach (i.e., using scheduling and assessment of emotions during pleasurable activities; Dimidjian et al., 2021). The second is to teach direct skills that enhance experiences, including present-moment awareness from Acceptance and Commitment Therapy and savoring from positive psychology (Bryan et al., 2022; Stone and Schmidt, 2022). Clinicians use both skills to enhance pleasurable experiences without substances, thus reducing the need to use substances to promote engagement in activities.

### Relationships

The PERMA model further suggests that fostering meaningful relationships with other individuals contributes to an authentically happy life (Seligman, 2011). Although substance use can cause strained social relationships (Daley, 2013), substances may facilitate social connections via two routes. First, individuals may use substances to help them manage social situations better (e.g., reduce anxiety or promote confidence). Alcohol, for example, is frequently used in social situations to aid individuals in their interactions with others (Collins et al., 1985; Monahan and Lannutti, 2000). Second, and most importantly, is the sense of community and belongingness that using substances provides and the culture of using certain substances with which people eventually identify (Moshier et al., 2012; Calvo et al., 2016). Many people make friends who share their use of substances and then use substances as bonding experiences (Dinges and Oetting, 1993; Donohew et al., 1999). Therefore, using substances provides a sense of community and belongingness and can make social interactions easier.

Relationships are already a focus of many treatments, as the risk of relapse and dangerous use of substances increases when individuals who use substances spend time with their friends or family who also use substances (Knight and Simpson, 1996; Falkin and Strauss, 2003). Clinicians may consider helping clients find a sense of community and belongingness beyond that of cultures and individuals involving substances so that the clients are not dependent on their needs to belong to a community of individuals who use the substances for which they are struggling to reduce use or abstain. Further, although substances can make social interactions easier, they do not address the underlying problem (e.g., social anxiety or low self-confidence). Skills and interventions to manage these uncomfortable emotions without using substances may help clients interact with people, thus reducing the need to use substances in social settings.

### Meaning

Meaning in the PERMA framework refers to the sense of purpose in one's life and the coherency of events that the person experiences (Seligman, 2011). Individuals can experience meaning from many different sources, including being in a family, having a career, and using skills or hobbies (Stone and Parks, 2018). Individuals may find meaning in their substance use, which is sometimes reported with mystical, spiritual, or religious interactions with substances (Krause et al., 2017). Further, the process of finding a supply, using the substances, which sometimes takes skills to use properly (i.e., properly injecting heroin or smoking methamphetamine), and doing activities during the high may provide a purpose in one's life. Many individuals with substance use disorder find that using substances becomes a significant purpose in their life (American Psychiatric Association, 2013), but, the problem is that this meaning and purpose is unfulfilling (Csabonyi and Phillips, 2020). Although they provide some purpose and meaning, this amount of time spent using and seeking substances limits individuals' ability to seek and engage in activities and causes that could provide a more significant and sustainable sense of meaning and purpose in their lives.

The basis for 12-step programs comes from building a sense of meaning and purpose with a higher power. Individuals in 12-step programs devote themselves to a higher power and confess to their high power about their shortcomings. Although some individuals succeed in 12-step programs, there are many limitations, such as the negative framing (Stone, in press), the religious component that does not engage everyone, and the questionable success outcomes (Flanagin, 2014). As such, we need more research on treatments that provide and encourage a sense of meaning and purpose beyond the 12-step strategy. For example, many positive psychological interventions, such as the Life Narrative or goal-setting (Stone and Parks, 2018), may be helpful, yet researchers have largely not tested these interventions on substance use disorders (Stone, in press). Still, if clinicians can help individuals experience meaning and purpose beyond substance use, then they may feel less of a need to use substances.

### Accomplishments

The final component of the PERMA model is achieving accomplishments that are of value to the individual (Seligman, 2011). Individuals can use substances to help them accomplish many different goals, including using amphetamines to get good grades in college (Varga, 2012), using alcohol to make friends (Collins et al., 1985; Monahan and Lannutti, 2000), and LSD to promote creativity (Janiger and De Rios, 1989); and some use these goals as reasons to continue or justify their use (DeSantis and Hane, 2010). Treating substance use disorders may be more challenging to treat if the substances themselves are also helping an individual function in some aspect (Bayard et al., 2004), such as in the case of self-medicating (Laroche et al., 2012), helping individuals at work or school (DeSantis and Hane, 2010), or are tied to religious experiences (Pahnke, 1967). Subsequently, substance use treatments may consider focusing on helping people function and achieve their goals while reducing or quitting their substance use.

Helping individuals achieve their goals and function without substances should focus on why individuals are struggling to function or achieve their goals. These treatments can vary and should focus directly on the client's presenting problem (e.g., if someone uses alcohol to make friends because they are anxious, then adding exposure to a substance use treatment may help resolve a justification for continuing to use alcohol). Further, many people may present with ambivalence about using substances to achieve their goals, for which a Motivational Interviewing approach may help resolve that ambivalence (Lundahl and Burke, 2009). Accordingly, if clinicians can treat the underlying use of the substances and help clients achieve their goals without substances, then the clients may not feel as strongly of a desire to use substances for those purposes.

## Discussion

### Why substances do not lead to an authentically happy life

If substances can provide engagement with all five components of the PERMA model, some might speculate as to why substance use does not lead to an authentically happy life; the answer is 2-fold: First, individuals use substances as temporary fixes to life problems, and substance use is not sustainable, which is why individuals continue to use substances repeatedly. Although substances can induce positive emotions, engage with pleasurable activities, and promote social connectedness, these experiences largely disappear when the substances are removed, unlike building non-substance-related activities and skills, which are sustainable. Secondly, although substances can provide these positive effects, they still come with many side effects, including expenses and homelessness, strained social relationships, physical health concerns, legal problems, and unemployment (Eyrich-Garg et al., 2008; Brook et al., 2009; Daley, 2013; Compton et al., 2014; Moore et al., 2020). Consequently, substances may provide temporary satisfaction with life, but they are not a sustainable solution and typically come with many adverse outcomes.

### Future directions

Given that this is the first article to apply the PERMA model to substance use, several viable future directions exist. For example, a study or studies that vigorously test the applicability and degree of fit between an individual who uses substances or someone with a substance use disorder's case conceptualization to the PERMA model could empirically test how applicable the PERMA model is to this population. Researchers may consider developing and validating a multidimensional scale of the PERMA model specifically for substance use. Further, it remains unclear if a treatment package designed to target the components of the PERMA model for substance use disorders would be effective or efficacious. Finally, more literature support for the PERMA model for substance use disorders may be beneficial, as this paper was limited in the depth of its literature review due to the paper format. A full-length review of this subject may provide further evidence as a foundation for future studies. Overall, there is much available work for researchers to do and many opportunities for future directions.

## Conclusion

The current article aimed to hypothesize and present research on why individuals use substances through the PERMA model and discuss how we can use this model in treatment. Although sparse and new, I found that the research supports the PERMA model as a conceptual framework for substance use. Although working toward satisfaction in all five of these domains may lead to an authentically happy and fulfilling life, when people use substances to reach these goals easier or quicker, it may have the potential to lead to dysfunctional substance use and substance use disorders. I want to encourage clinicians to use this framework in their conceptualization of their clients with substance use disorders and focus treatment on using non-substance-related alternatives to help build an authentically happy life without dependence on substances.

## Author contributions

The author confirms being the sole contributor of this work and has approved it for publication.

## Funding

Funding was supported as part of a professional development fund from the Medical University of South Carolina psychology pre-doctoral internship.

## Conflict of interest

The author declares that the research was conducted in the absence of any commercial or financial relationships that could be construed as a potential conflict of interest.

## Publisher's note

All claims expressed in this article are solely those of the authors and do not necessarily represent those of their affiliated organizations, or those of the publisher, the editors and the reviewers. Any product that may be evaluated in this article, or claim that may be made by its manufacturer, is not guaranteed or endorsed by the publisher.
